# Incidental Cutaneous Microcystic/Reticular Schwannoma in Pilonidal Sinus

**DOI:** 10.4274/balkanmedj.galenos.2019.2019.7.126

**Published:** 2019-12-20

**Authors:** Recep Bedir, Orhan Semerci, Gülname Fındık Güvendi

**Affiliations:** 1 Department of Pathology, Recep Tayyip Erdoğan University School of Medicine, Rize, Turkey

Schwannoma, a non-malign peripheral nerve sheath tumor, develops slowly and is usually clinically inapparent. It usually arises after the fourth decade of life in the subcutaneous tissue of the extremities and in the head and neck region of patients, with no gender bias. Microcystic/reticular schwannoma (MRS) is a rare histological form of schwannoma, first described in 2008 by Liegl et al. (1) MRS is a recent addition to this group of tumors, which shows predilection for visceral organs, without Antoni A and Antoni B areas or Verocay bodies (2,3). Cutaneous presentation of MRS is very unusual neoplasm which seven cases reported in the sources until to date (4). Here we report a case of incidental cutaneous MRS in a pilonidal sinus material.

A 28-year-old male patient was operated on for the presence of pilonidal sinus. Macroscopically there was a fistula with a hairy body in the pilonidal sinus specimen beneath a gray-white solid mass with focal myxoid patches located under the skin. The diameter of the lobular mass was 2x1.5 cm, which was well segregated. Microscopically, the cells  were spindle-like neoplastic cells with eosinophilic cytoplasm and hyperchromatic nuclei. There was no mitoses, necrosis or nuclear atypia. The tumor cells were lace-like, retiform or pseudoglandular with heavy myxoid matrix (Figure 1 a-c). Immunohistological staining of the sections showed intense and diffuse staining for, vimentin, S-100 and glial fibrillary acidic protein (GFAP). No staining was evident for pan-CK(AE1/AE1), CD34, HMB-45 and epithelial membrane antigen (Figure 1 d-f). The final diagnose was cutaneous MRS. Written informed consent was obtained from the patient.

Schwannomas are benign mesenchymal tumors that originate from  the Schwann cells forming the neural sheath, with multiple  morphological sub-types, including cellular, ancient, plexiform, epithelioid, glandular, melanotic, MRS and hybrid schwannoma/perineurioma (1,4). MRS is a unique variant of schwannoma with benign biological behavior. Differential diagnosis of MRS localized in the skin includes lymphangioma, nerve sheath myxoma, reticular perineuroma, cutaneous lipomatous neurofibroma, a myxoid variant of cellular neurothekeoma and extraskeletal myxoid chondrosarcoma (4). Immunohistochemistry is important in the differential diagnosis of MRS. The majority of tumors in the literature  is also positive  for vimentin, S-100  and GFAP (3).

In conclusion the reticular growth pattern and myxois matrix is very important for distinguish the other soft tissue tumors from cutaneous MRS.

## Figures and Tables

**Figure 1:**
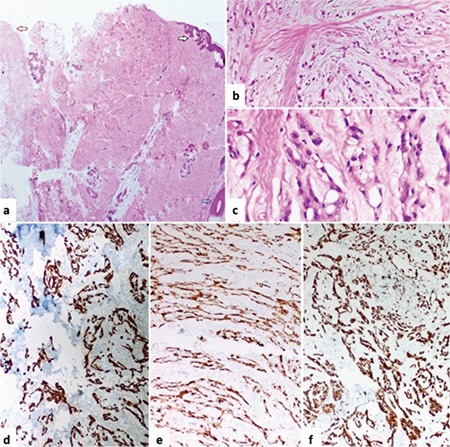
(a) The tumor (red arrow) was located under the skin (black arrow). There is also a hair follicle in the deep dermis (blue arrow) (H&Ex40) (b) The tumor shows numerous microcystic structures formed by spindle-shaped neoplastic cells with eosinophilic cytoplasm. (H&Ex200) (c) Tumor was forming lace-like, retiform or pseudoglandular structures which contain heavy myxoid matrix (H&Ex400). (d) Tumor showed diffuse positivity for S100 (x200) e) Tumor showed diffuse positivity for GFAP (x200). (f) Tumor showed diffuse positivity for vimentin (x200).
